# X-ray irradiation activates K^+^ channels via H_2_O_2_ signaling

**DOI:** 10.1038/srep13861

**Published:** 2015-09-09

**Authors:** Christine S. Gibhardt, Bastian Roth, Indra Schroeder, Sebastian Fuck, Patrick Becker, Burkhard Jakob, Claudia Fournier, Anna Moroni, Gerhard Thiel

**Affiliations:** 1 Department of Biology, Membrane Biophysics, Technische Universität Darmstadt, Schnittspahnstrasse 3, 64287 Darmstadt, Germany; 2 Department of Biophysics, GSI Helmholtzzentrum für Schwerionenforschung GmbH, Planckstrasse 1, 64291 Darmstadt, Germany; 3 Department of Biosciences and CNR IBF-Mi, Università degli Studi di Milano, Via Celoria 26, 20133 Milano, Italy

## Abstract

Ionizing radiation is a universal tool in tumor therapy but may also cause secondary cancers or cell invasiveness. These negative side effects could be causally related to the human-intermediate-conductance Ca^2+^-activated-K^+^-channel (hIK), which is activated by X-ray irradiation and affects cell proliferation and migration. To analyze the signaling cascade downstream of ionizing radiation we use genetically encoded reporters for H_2_O_2_ (HyPer) and for the dominant redox-buffer glutathione (Grx1-roGFP2) to monitor with high spatial and temporal resolution, radiation-triggered excursions of H_2_O_2_ in A549 and HEK293 cells. The data show that challenging cells with ≥1 Gy X-rays or with UV-A laser micro-irradiation causes a rapid rise of H_2_O_2_ in the nucleus and in the cytosol. This rise, which is determined by the rate of H_2_O_2_ production and glutathione-buffering, is sufficient for triggering a signaling cascade that involves an elevation of cytosolic Ca^2+^ and eventually an activation of hIK channels.

In recent years, it became evident that K^+^ channels play an important role in the regulation of cell differentiation. Some of the main targets of K^+^ channel activity in this context are the control of the cell cycle^1^,^2^,^3^ and the induction of apoptosis^3^,^4^,^5^,^6^,^7^; also a role of K^+^ channels in cell invasion is well documented^8^,^9^,^10^. With the emerging awareness of a role of K^+^ channels in the regulation of cell differentiation it was interesting to find that exposure of cells to ionizing irradiation (IR) triggered the activation of the human-intermediate-conductance Ca^2+^ activated K^+^ channel (hIK). This response was rapid and occurred within minutes after stressing cells with low dose X-ray; e.g. doses, which are conventionally used in cancer treatment. The response of K^+^ channels to IR stress turned out to be cell- specific and was most evident in cells, which functionally expressed hIK channels and in which hIK activity was low before IR. The established role of hIK channels in cell proliferation^11^,^12^,^13^,^14^ and migration^8^,^9^,^10^,^15^ together with the results of experiments in which hIK channels were specifically blocked, suggested that an irradiation-induced elevation of hIK activity has important impacts on cell differentiation. It was found that inhibition of hIK channels by specific blockers like Clotrimazole and Tram-34 slowed cell proliferation and cell migration. Ionizing irradiation in turn stimulated the latter process via its activation of hIK channels. These data stress an indirect radio-sensitivity of hIK channels with an impact on cell differentiation^16^.

In previous experiments, it was already found that an activation of hIK channels by IR was suppressed when the cytosolic Ca^2+^ buffer concentration was elevated^16^. The results of these experiments suggested that IR stimulates a rise in the concentration of cytosolic free Ca^2+^ (Ca^2+^_cyt_) and that the latter activates hIK channels. The complementary finding that an application of extracellular H_2_O_2_ caused an increase in Ca^2+^_cyt_ furthermore suggested that an intracellular rise of radicals is the primary step in a signal cascade, which eventually results in a rise in Ca^2+^_cyt_. Here we examine whether IR of cells with X-rays or micro-irradiation with UV laser indeed cause an elevation of free radicals in cells. Using the H_2_O_2_-sensitive reporter protein HyPer we find that both types of irradiation stress cause a rapid elevation of H_2_O_2_ not only in the nucleus but also in the cytosol. Micro-irradiation with laser light showed that irradiation of the nucleus generated more radicals than the same treatment of the cytosol. Live measurements of single cells after X-ray irradiation highlighted a long lasting increase of the amount of H_2_O_2_ throughout the entire cell. The use of another ratiometric sensor, which is measuring the glutathione redox potential, shows that the dynamics in the increase in H_2_O_2_ concentration is determined by an ongoing production and buffering by glutathione.

## Results

### Recording of H_2_O_2_ in cells

H_2_O_2_ is one of the major oxygen free radical species (ROS), which is generated in cells in response to stress. Its concentration can be monitored in cells with high spatial and temporal resolution by the genetically encoded sensor HyPer. This fusion product of a fluorescent protein and a cysteines containing transcription factor from bacteria reacts specifically with peroxide, which in turn alters the fluorescent properties of the sensor^17^. To calibrate the HyPer signal the sensor was transiently expressed in HEK293 cells and these cells were then incubated in 400 μL PBS buffer. 100 μL of a H_2_O_2_ containing solution was added and mixed with the PBS buffer to give final concentrations between 10 μM and 200 μM in a constant volume of 500 μL incubation buffer.

**Figure 1:**
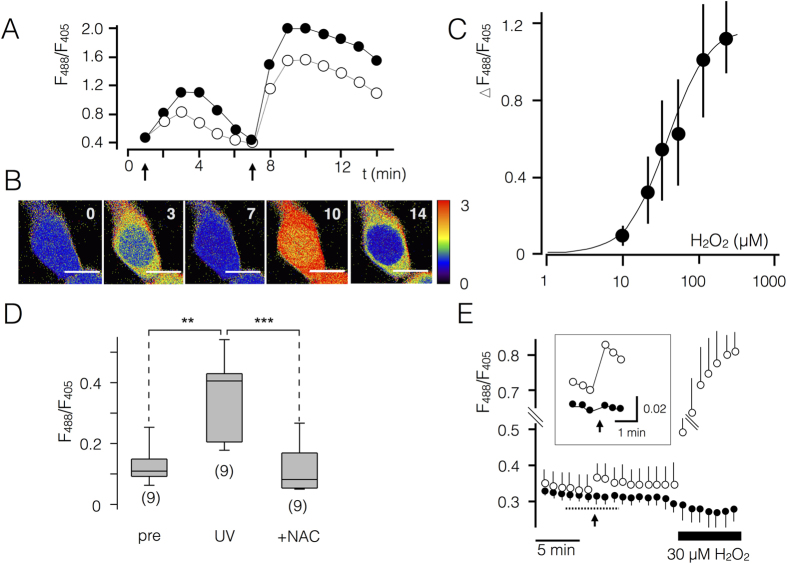
Characterization of HyPer sensor for radiation stress. (**A**) Fluorescence ratio F_488_/F_405_ from HyPer in cytoplasm (closed symbols) and nucleus (open symbols) in a HEK293 cell in response to different concentrations of external H_2_O_2_. The arrows indicate addition of 20 μM (left arrow) and 100 μM (right arrow) to external solution. Rise in HyPer signal following H_2_O_2_ addition is transient; H_2_O_2_ concentration was further increased once the signal had decayed back to resting level. The corresponding pseudo color images of an exemplary cell challenged with H_2_O_2_ are shown in (**B**). Experiments as in (**A**) were used for the calibration curve for HyPer (n = 4–20 ± SD). The increase in the ratio over the background (ΔF_488_/F_405_) is plotted as a function of the extracellular H_2_O_2_ concentration. Fitting the data (line) with a sigmoidal-function (eq. 1) yields a concentration of H_2_O_2_ for half-maximal increase (K_ox_) in the F_488_/F_405_ ratio of 37 μM. (**D**) F_488_/F_405_ ratio of HEK293 cells measured before (pre), after micro-irradiation with 3 μJ/μm^2^ 405 nm laser (UV) and after treating the same cells with H_2_O_2_ scavenger N-acetylcysteine (6 mM) in the bath medium (+NAC). (**E**) Laser micro-irradiation has no effect on the pH of the cytosol. HEK293 cells transiently expressing HyPer (open symbols) or the H_2_O_2_ insensitive mutant SypHer (closed symbols) were micro-irradiated at arrow (3 μJ/μm^2^, 405 nm laser) and subsequently treated with 30 μM H_2_O_2_ (solid bar). Micro-irradiation and H_2_O_2_ addition to the external medium trigger an increase in F_488_/F_405_ ratio (n = 3, ± SD). The corresponding ratio remains stable after micro-irradiation and fails to increase in response to the elevation of external H_2_O_2_ (n = 4 ± SD). Data taken during time indicated by dotted line are magnified in inset. Number in images in (**B**) provide time in min for duration of experiment and correspond to data in (**A**). Scaling of pseudo colors is shown with images; numbers denote the minimum (blue = 0) and maximum (red = 3) F_488_/F_405_ ratio. Scale bar in images: 10 μm.

Representative false color images for the ratio of F_488/405_ and the corresponding ratios of the HyPer signal in cytoplasm and nucleus are shown in Fig. 1A,B for one cell before and after adding H_2_O_2_ to the bath medium. The data show that addition of H_2_O_2_ causes a rise in the HyPer ratio over 2 to 3 min; the latter presumably reflects an efficient buffering of H_2_O_2_ in the cells. The H_2_O_2_ induced change in the HyPer ratio is the consequence of an inverse change in the fluorescence at F_405_ and F_488_ nm (Fig. S1A).

A subsequent increase of the external H_2_O_2_ concentration caused a further rise of the HyPer signal, which was again reduced by buffering (Fig. 1A,B). From a large number of similar experiments we constructed an *in vivo* calibration curve for the HyPer ratio as a function of the external H_2_O_2_ concentration (Fig. 1C). The data were fitted with a sigmoidal function (eq. 1) to estimate the concentration of H_2_O_2_ for a half-maximal (K_ox_) increase in the F_488/405_ ratio.


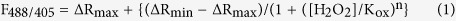


In this equation ΔR_min_ and ΔR_max_ represent the minimal and maximal change in F_488/405_ ratio respectively. Fitting of the experimental data yields a half-maximal value K_ox_ for a concentration of 37 μM H_2_O_2_ in the external solution with a value of 1.6 for the factor n. The calibration curve is similar to results from a recent report, which shows in HEK293 cells a dynamic range for the HyPer sensor between 1 μM and 50 μM H_2_O_2_ in the external medium^18^. Considering the low permeability of H_2_O_2_ across membranes and the buffering of H_2_O_2_ inside the cells we must assume that the actual concentration of the molecule inside a cell may be much lower than that in the external medium. An *in vitro* calibration of HyPer reports a K_ox_ value of 160 nM^17^ e.g. a value, which is 200 times smaller than that of the *in vivo* approach. The discrepancy between the *in vivo* and *in vitro* calibration is consistent with reports, which shows a 200^18^,^19^ or even up to 650^20^ fold concentration gradient between H_2_O_2_ added to the extracellular side and the corresponding concentration of the molecule inside a cell. Taken together this means that the calibration curve in Fig. 1C is in a strict sense only valid for the cytoplasm of HEK293 cells and may be different in another cellular compartment or in other types of cells.

To test whether the rise in the HyPer ratio after IR is indeed due to a generation of H_2_O_2_, nine HEK293 cells were first micro-irradiated with the 405 nm laser (3 μJ/μm^2^) in PBS buffer. After reaching a maximal rise in the HyPer signal, which generally occurred 30 s to 1 min after irradiation, cells were treated with 6 mM N-Acetylcysteine (NAC) (Fig. 1D). The cell-permeable antioxidant NAC effectively scavenged the rise in HyPer signal, which was elicited by UV-irradiation (Fig. 1D). When cells were stimulated by UV-micro-irradiation in the presence of 10 mM NAC in the external buffer the HyPer signal remained unaffected (data not shown). The results of these experiments confirm that the rise in the HyPer signal reflects the rise of H_2_O_2_ in cells after irradiation stress.

Since HyPer exhibits also some pH-sensitivity it is necessary to test whether changes in the pH contribute to the UV triggered HyPer signal. For this purpose the H_2_O_2_-insensitive HyPer-variant SypHer^21^ was employed. In SypHer the critical cysteine at position 199 of the OxyR domain is changed into a serine, creating a ratiometric sensor with the same pH sensitivity of HyPer, but with no sensitivity to H_2_O_2_ (Fig. 1E). Micro-irradiating HEK293 cells, which transiently express SypHer, with the 405 nm laser (3 μJ/μm^2^) had no effect on the fluorescence ratio (Fig. 1E). Hence UV-irradiation has no impact on the pH of cells; the increase in the HyPer signal, which we observe after laser micro-irradiation, only reports a generation of H_2_O_2_ in the stressed compartment.

### UV-micro-irradiation elicits a rapid generation of H_2_O_2_ in nucleus and cytosol

**Figure 2:**
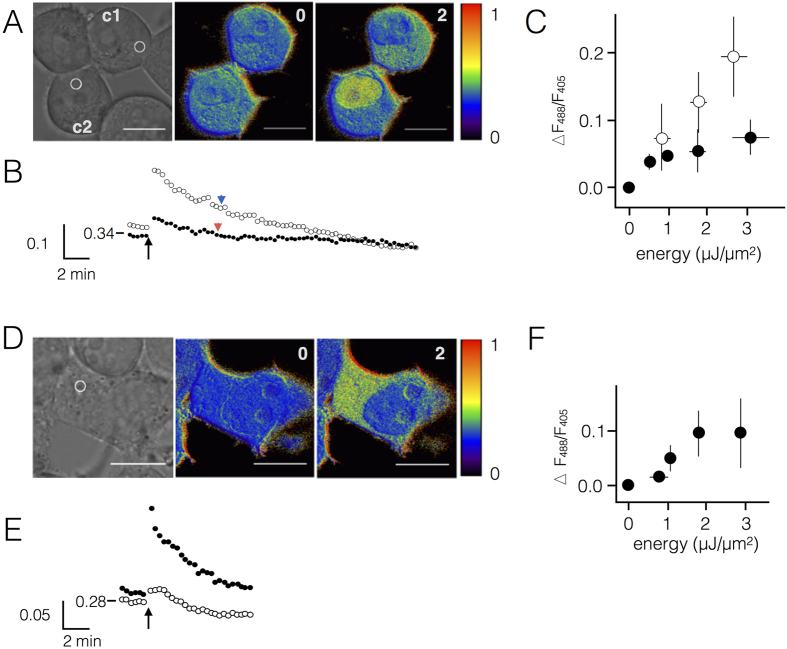
UV-laser micro-irradiation triggers in mammalian cells a generation of H_2_O_2_ in nucleus and cytoplasm. (**A**) Exemplary A549 cells (c1, c2), which express H_2_O_2_ sensor HyPer, were imaged and irradiated on a confocal microscope with a 405 nm laser (3 μJ/μm^2^) in the cytoplasm (c1) or in the nucleus (c2). The positions of micro-irradiation are indicated by circles in microscopic images (left panel). Pseudo-color images of F_488_/F_405_ ratio before and after micro-irradiation show increase in the HyPer signal in compartment, which was irradiated. (**B**) F_488_/F_405_ ratio from a region of interest (ROI) in cytoplasm (c1, closed symbols) and nucleus (c2 open symbols) from cells in (**A**) before and after micro-irradiation (at arrow). The arrowheads indicate half time of recovery. (**C**) Mean (±SD) maximal increase in ΔF_488_/F_405_ ratio in nucleus (open symbol, n ≥ 4) or cytoplasm (closed symbol, n ≥ 11) after irradiating respective compartments with different energies of laser light. (**D**) Same as in (**A**) but with a HEK293 cell irradiated in the cytoplasm (2 μJ/μm^2^, 405 nm, circle in left panel). Pseudo-color images before and after micro-irradiation show a strong increase in F_488_/F_405_ ratio in the cytoplasm. (**E**) Dynamics of F_488_/F_405_ ratio from cytoplasm (closed symbols) and nucleus (open symbols) from cell in (**D**) before and after micro-irradiation; time of irradiation indicated by arrow. (**F**) Mean (±SD) maximal increase in ΔF_488_/F_405_ ratio in cytoplasm (closed symbol) of HEK293 cells after irradiating the respective compartment with different energies of laser light (n ≥ 4). Numbers in images give time in min after irradiation. Vertical calibration bars show ratio F_488_/F_405_ for HyPer. Scaling of pseudo colors is shown with images; numbers denote the minimum (blue) and maximum (red) F_488_/F_405_ ratio. Scale bar in images: 10 μm.

Circumstantial evidence suggests a scenario in which IR generates in A549 cells H_2_O_2_ as an early event in a signaling cascade, which finally triggers activation of hIK channels^16^. To test this hypothesis and to unravel the spatial/temporal dynamics of the radiation-triggered signaling cascade we micro-irradiated cells with a 405 nm UV-laser. In combination with confocal microscopy this allows monitoring of the distribution of H_2_O_2_ in individual cells after IR. It is well established that UV-laser micro-irradiation is creating radicals in the nucleus by an ill understood mechanism and that the latter cause DNA double-strand breaks^22^,^23^,^24^,^25^. It was also reported that irradiation of cells with UV-A light triggered an elevation of ion channel activity, which was presumably mediated by a generation of H_2_O_2_^26^,^27^. In the context of an activation of ion channels in the plasma membrane the compartment-specific micro-irradiation of either nucleus or cytoplasm should show whether radicals, which are produced in the nucleus are diffusing into the cytoplasm for an activation of hIK channels or whether an irradiation of the cytosol alone is already sufficient for the generation of radicals. To monitor the putative generation of H_2_O_2_ in response to UV-irradiation we measured excursions in the concentration of this radical in the nucleus and in the cytoplasm of cells. Hydrogen peroxide is a long-lived radical species and an end product of many short-lived radicals. A549 cells, which expressed the fluorescent H_2_O_2_ sensor protein HyPer^17^ were therefore challenged with 405 nm laser light. An example for a local UV-irradiation with 3 μJ/μm^2^ in two A549 cells, one irradiated in the nucleus, the other in the cytoplasm, is depicted in Fig. 2A–C. This treatment caused in the cell, which was irradiated in a small volume of the nucleus, an immediate rise in the HyPer signal throughout the entire nucleus (supplements Fig. S2A,B). Important to note is that the signal was confined to the nucleus, i.e. in the irradiated cellular structure (Fig. S2A). The cell, which was irradiated in the cytoplasm, also responded with a rapid rise in the fluorescent signal in the cytoplasm (Fig. 2A,B). The signal again remained confined to the irradiated compartment (supplements Fig. S2B). Control experiments presented in Fig. 1 have shown that the rise in the HyPer signal is exclusively caused by an increase in H_2_O_2_; it is not due to UV irradiation-induced pH changes.

The mechanism by which UV-A light causes the production of radicals in cells is still poorly understood and generally explained by an activation of endogenous photosensitizers^28^,^29^. This raises the question whether the potency of UV light in triggering H_2_O_2_ production in the cytosol and the nucleus is augmented by the HyPer sensor. To test whether the HyPer chromophore acts as an artificial photosensitizer we examined the UV induced increase in H_2_O_2_ as a function of the absolute HyPer fluorescence. In the case that HyPer acts as photosensitizer a given UV micro-irradiation should cause in different cells an increase in H_2_O_2_ concentration as a function of the HyPer concentration; the latter is reflected by the absolute fluorescence at 405 nm. An analysis of the maximal rise of the ratio ΔF_488_/F_405_ following UV micro-irradiation with 3 μJ/μm^2^ as a function of the HyPer fluorescence is shown in supplements Fig. S3. The data reveal no apparent correlation between the two parameters suggesting that HyPer does not act as a photosensitizer.

Collectively the results from these experiments show that UV-irradiation triggers the development of H_2_O_2_ in the nucleus and in the cytoplasm. The signal is strongest in the compartment, which was primarily stimulated by UV light; the nuclear envelope is a moderate diffusion barrier so that diffusion of H_2_O_2_ out of the nucleus into the cytoplasm seems irrelevant for an activation of channels in the plasma membrane.

The same procedure was repeated in 21 and 80 cells in which the nucleus or cytoplasm respectively was irradiated with a range of laser energies from 0.7 to 2.9 μJ/μm^2^. The data in Fig. 2C show that the mean maximal elevation of the HyPer signal (ΔF_488_/F_405_) in the nucleus and in the cytoplasm is a function of the laser energy, which was used to challenge the cell compartments. The results of these experiments show that an approximately two-fold higher laser energy dose is necessary to generate in the cytoplasm the same amount of H_2_O_2_ as in the nucleus (Fig. 2C). An increase in the HyPer ratio of 0.1 in the cytosol of cells in Fig. 2A,B corresponds according to our calibration to a treatment of cells with 9 μM H_2_O_2_ in the external medium. Using an *in vitro* calibration with purified HyPer protein^17^ this translates into an internal H_2_O_2_ concentration of 40 nM.

The different amplitudes of the UV-triggered HyPer signals in cytoplasm and nucleus could depend on two reasons. The most straightforward explanation would be that the same dose of UV-irradiation causes a lower increase of H_2_O_2_ concentration in the cytosol compared to the densely packed nucleus. An alternative explanation could be that the emerging radicals are more rapidly buffered in the cytosol than in the nucleus. Interesting to see is that the HyPer signals decay after stimulation in an exponential fashion. As a first approximation we can assume that this reflects the buffering of H_2_O_2_ in the respective compartments. In the case that the lower signal amplitude after UV irradiation in the cytosol is due to a more efficient buffering of H_2_O_2_ the signal should relax faster in the cytosol than in the nucleus. For a test of this hypothesis we estimated the half times for the relaxations of the signals from cytosol and nucleus (Fig. 2B). The representative data show that both signals decay with about the same velocity. The difference in the signal in the two compartments most likely reflects different amplitudes in the transient increase in H_2_O_2_ in the two cell compartments.

Next we examined whether the UV light induced generation of H_2_O_2_ is cell type specific. In previous experiments, it was found that an irradiation-induced elevation of the membrane conductance was observed in A549 cells but not in HEK293 cells. This cell specificity was attributed to the presence or absence of hIK channels in the responsive A549 cells versus the non-responsive HEK293 cells^16^. This explanation is however not ruling out the possibility that the cell-specific response to radiation stress is caused by differential signaling properties upstream of the hIK channel. To test the possibility that the two cell lines respond differently HyPer was expressed in HEK293 cells and the latter cells were micro-irradiated in the cytoplasm by UV-light. The data in Fig. 2D–F show that the same treatment causes a rapid elevation of the cytoplasmic H_2_O_2_ concentration also in HEK293 cells. The increase in the ratio of the H_2_O_2_ reporter in the cytoplasm for a reference energy of 3 μJ/μm^2^ irradiation is not significantly different (p = 0.2) between the two types of cells (Fig. 2C,F). The results of these experiments show that the irradiation-induced signaling cascade is not appreciably different between the two types of cells. Both types of cells generate an equivalent amount of H_2_O_2_ after irradiation stress.

### Monitoring H_2_O_2_ generation in response to X-ray irradiation

**Figure 3:**
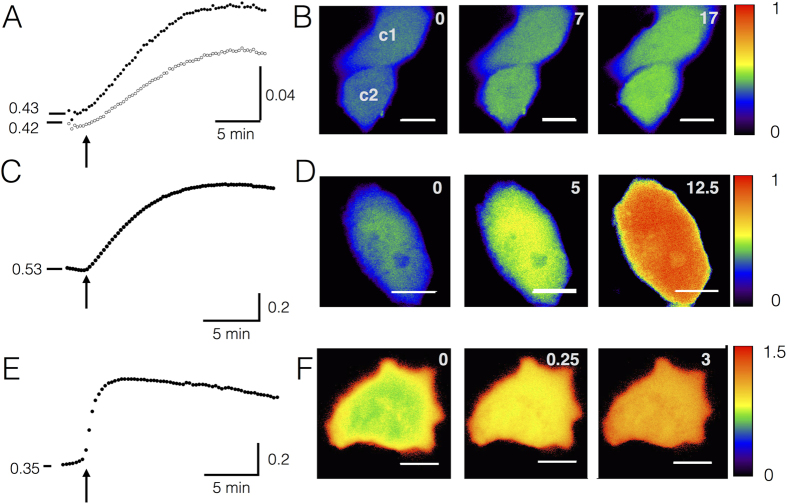
Irradiation of mammalian cells with X-rays triggers generation of H_2_O_2_ and redox buffering. F_488/405_ ratio of HyPer in two A549 cells (c1, c2) before and after exposing them to X-ray with 1 Gy in (**A**) or 10 Gy in (**C**). The corresponding pseudo-color images of cells before and after irradiation are shown in (**B**) and (**D**) for 1 and 10 Gy treatment respectively. The colors range from blue (F_488/405_ = 0) to red (F_488/405_ = 1). (**E**) Exemplary A549 cell, which transiently expressed the glutathione redox-sensor Grx1-roGFP2, exhibits a rapid increase in the fluorescence ratio F_405_/_488_ after irradiation with 10 Gy of X-rays; this reports an oxidation of glutathione redox buffer. The corresponding pseudo-color images taken before and after irradiation stress are shown in (**F**). Vertical calibration bars show ratio F_488_/F_405_ for HyPer and F_405_/F_488_ nm for Grx1-roGFP2. The colors range from blue (F_405_/F_488_ = 0) to red (F_405_/F_488_ = 1.5). Numbers in images denote time in min after irradiation. Scale bar in images: 10 μm.

**Figure 4:**
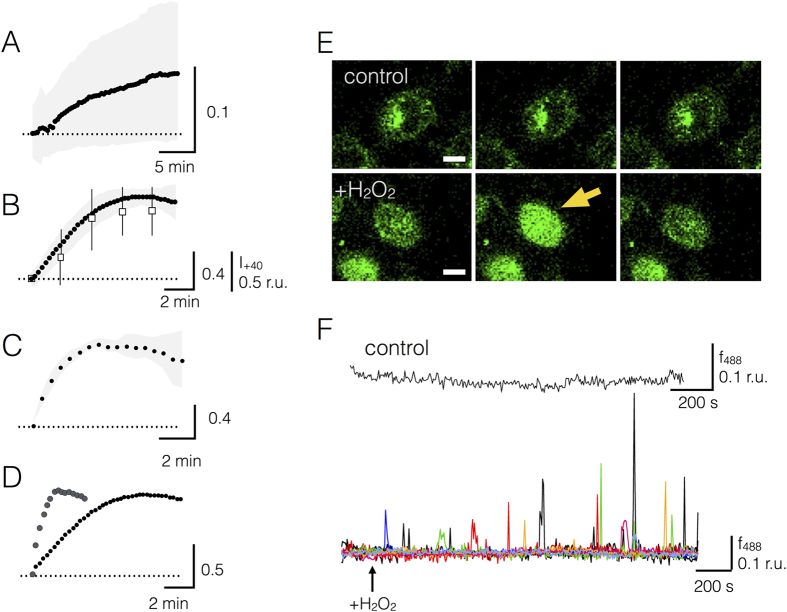
Mean increase in X-ray stimulated rise in H_2_O_2_, redox buffering and H_2_O_2_ stimulate increase in Ca^2+^_cyt_. Mean increase in ΔF_488_/F_405_ ratio from HyPer over background in response to 1 Gy (**A**) or 10 Gy (**B**) X-ray irradiation. Means ± SD from n = 38/N = 4 and n = 35/N = 3 recordings. Open squares in (**B**) show the mean relative increase (±SD) in instantaneously activating current in relative units (r. u.) at + 40 mV (I_+40_) from eight A549 cells after start of irradiating cells with 10 Gy X-ray. The maximal excursion in current of each cell was normalized to the mean maximum of the HyPer signal. (**C**) Mean increase in ΔF_405_/F_488_ nm ratio from Grx1-roGFP2 over background in response to 10 Gy X-ray irradiation. Means ± SD from n = 15/N = 2 recordings. In (**D**) the mean signals for H_2_O_2_ (solid symbols, form (**B**)) and for the redox buffer (data from c) in response to 10 Gy X-ray are scaled to the same ordinate. (**E**) Representative images of Fluo-4 fluorescence in A549 cells in absence (top row, control) and presence (bottom row, +H_2_O_2_) of 3 μM H_2_O_2_ in external medium. The arrow in the lower row shows a cell, which reveals a transient rise in Ca^2+^_cyt_ 60 sec after challenging cells with H_2_O_2_. Images taken with time gaps of 20 and 35 sec respectively. (**F**) Continuous recording of Fluo-4 fluorescence (f488) from an exemplary control cell (top) and in an overlay of 8 cells challenged with 3 μM H_2_O_2_ in the bath medium (bottom, each cell is shown in a different color). H_2_O_2_ was added at time indicated by arrow. Fluorescence signals were obtained by subtracting background fluorescence. Scale bar = 10 μm.

Micro-irradiation has the advantage that the cells can be stimulated with high precision and subsequently monitored with a high spatial/temporal resolution. A disadvantage is the difficulty to define the local dose of a UV-light and to compare the data with X-ray irradiation^30^. Notably UV-light has a lower energy than X-rays and is unlike the latter not sufficient for hydrolyzing water molecules. Since both electromagnetic waves are also absorbed by different molecules it is obvious that X-rays and UV-light will generate different types of radicals in cells. To test the effect of X-rays on the dynamics of H_2_O_2_ development directly, A549 cells were irradiated with 1 Gy or 10 Gy X-ray and the HyPer fluorescence was directly monitored on a microscope, which was coupled to an X-ray source. Representative data in Fig. 3A–C show that both doses of X-ray elicited a fast rise in the HyPer signal throughout the entire cell. A similar rise in the HyPer signal was recorded in a large number of A549 cells challenged with the two doses of X-ray (Fig. 4A,B). The mean response of 38 cells (N = 4) to 1 Gy revealed a large variability in amplitude and kinetics while cells responded with a larger increase and with a more similar kinetics to 10 Gy X-ray stimulation. Similar responses were also elicited by the same treatment of HEK293 cells where irradiation with 1 and 10 Gy X-ray triggered a mean maximal increase in the HyPer ratio of 0.06 + 0.03 and 0.4 + 0.27 respectively. The results of these experiments suggest that the generation of H_2_O_2_ is a genuine response of cells to X-ray stress and not specific for A549 cells.

### Consequences of H_2_O_2_ generation

In relation to the activation of hIK channels by X-ray^16^ it is important to note that already 1 Gy X-ray causes a measurable elevation of the H_2_O_2_ concentration. This is according to the calibration curve from Fig. 1C equivalent to what will be generated by treating cells with low micromolar concentrations of H_2_O_2_ in the external solution. Hence a micromolar concentration of H_2_O_2_ in the external medium should be sufficient for elevating the free concentration of Ca^2+^ in the cytosol and for stimulating the Ca^2+^_cyt_ sensitive hIK channels in A549 cells. Previous experiments have shown that the latter is indeed the case. A treatment of A549 cells with 3 μM H_2_O_2_ in the external medium was able to augment hIK activity in these cells^16^.

To test whether 3 μM H_2_O_2_ in the external medium is also sufficient for elevating Ca^2+^_cyt_ e.g. the signal upstream of hIK channel activation, we loaded A549 cells with Fluo-4 and recorded the fluorescence in untreated control cells and in cells exposed to 3 μM H_2_O_2_ in the external medium. The data in Fig. 4E,F show that the Fluo-4 signal, a reporter for Ca^2+^_cyt_, is stable in untreated control cells; the same long term stability was obtained in more than 100 control cells. Addition of H_2_O_2_ to the bath in contrast triggered in 25% of the cells detectable spikes or oscillations of Ca^2+^_cyt_, which exceeded two times of the standard deviation of the noise. Figure 4E,F depicts exemplary H_2_O_2_-triggered oscillations in eight exemplary cells. The results of these experiments confirm the prediction that 3 μM H_2_O_2_ in the external medium, e.g. a treatment, which causes the same elevation of intracellular H_2_O_2_ as 1 Gy X-ray, is sufficient to trigger an elevation in the Ca^2+^_cyt_ concentration. Hence stressing cells with 1 Gy X-ray can activate hIK channels via a signal transduction cascade, which involves an elevation of H_2_O_2_ and a subsequent rise in Ca^2+^_cyt_; the latter is the known positive modulator of hIK channels^31^.

To further test the causal relationship between the kinetics of X-ray induced radicals and an activation of hIK channels, membrane currents of A549 cells were recorded before and immediately after exposing cells to 10 Gy X-ray. We reasoned that an X-ray induced activation of hIK channels should reflect the kinetics of H_2_O_2_ elevation, which is induced by the same treatment. The data in supplements Fig. S4 show the current response of an exemplary A549 cell to a test voltage step from −60 to +40 mV before as well as 6 min after start of irradiating the cell with 10 Gy X-ray. It occurs that the instantaneous activating conductance is strongly augmented by this treatment. The corresponding current/voltage (I/V) relation also shows that the irradiation induced conductance causes a negative shift of the reversal voltage (mean −31 ± 9 mV, n = 6); this hyperpolarization is in agreement with previous data, which have shown that the irradiation-triggered current in A549 cells is at least in part carried by the hIK channel^16^. To further test this hypothesis we treated cells after an exposure to 10 Gy X-ray with 300 nM TRAM-38, a specific inhibitor of hIK channels^32^. The exemplary data in the supplements (Fig. S5) show that TRAM-38 reverses the X-ray induced current concomitant with a positive shift of the reversal voltage. Collectively these data, which were confirmed in 3 cells, underscore that X-ray irradiation augments the activity of hIK channels in A549 cells.

To compare the kinetics of the two events i.e. H_2_O_2_ generation and hIK activation we plotted the instantaneous activating current at +40 mV of eight A549 cells as a function of time after challenging them with 10 Gy X-ray. For comparison of the data sets the maximal excursion of the current was normalized to the maximum of the mean HyPer signal. The data in Fig. 4B show that irradiation of the cells results in an elevation of a membrane conductance with a kinetics, which resembles that of the increase in H_2_O_2_. As a final test for a causal relation between radiation-triggered increase in H_2_O_2_ and channel activation we measured membrane currents in A549 cells before and after adding a high saturating concentration of H_2_O_2_ to the external medium. This caused the expected elevation of an instantaneously activating channel. A subsequent treatment of the same cell with the radical scavenger Tempol^33^ reverted the activating effect of H_2_O_2_ (Fig. S4C,D) The results of these experiments, which were confirmed with three additional cells (Fig. S4D), show that H_2_O_2_ is able to augment the activity of an instantaneous activating channel and that H_2_O_2_ buffering is able to invert it.

### Relationship between H_2_O_2_ generation and its buffering by glutathione

Radicals, which emerge in the cytosol or in the nucleus during physiological reactions or stress are generally removed by efficient buffers^34^. To examine the contribution of redox-buffers to the kinetics of signal processing during radiation stress we also monitored the redox status of glutathione, the prominent buffer of radicals in cells, with the highly selective ratiometric sensor Grx1-roGFP2^35^,^36^. A549 cells, which express the fluorescent sensor, were therefore imaged with the same set up used in Fig. 3A–D. The exemplary data in Fig. 3E,F show that irradiation of A549 cells expressing the glutathione redox-sensor triggered a fast increase in the sensor signal. The massive and rapid increase in the amount of oxidized glutathione is the result of a fast buffering of various radical species, which were generated by X-ray radiation. An increase in glutathione redox-potential, which results from elevated ROS levels, were also observed after irradiation with 1 Gy X-ray. The results of these experiments are in good agreement with data from Fig. 3A–D and confirm in an independent manner the generation of radicals in response to X-ray irradiation.

The robust increase in the Grx1-roGFP2 and HyPer signals following irradiation with 10 Gy X-ray allows an estimation of the kinetics of the two responses. The exemplary data show that the rise of both signals can be, as a first approximation, fitted by single exponentials. This yields a mean τ value of 4.2 ± 1.6 min (n = 35; N = 3) for the rise in H_2_O_2_ and of 0.9 ± 0.5 min for the filling of the redox buffer (n = 15; N = 2). The result of this analysis and plotting the two responses in one graph (Fig. 4D) shows that the concentration of H_2_O_2_ rises slower than the redox buffer decreases. A reasonable explanation for the dynamics of the two signals implies that the rise in HyPer signal is determined by H_2_O_2_ generation and buffering.

## Discussion

It has frequently been speculated that ionizing irradiation but also UV-A light trigger in cells a rise of intracellular ROS and that the latter act as messengers in signaling cascades^26^,^27^,^37^,^38^,^39^ including Ca^2+^_cyt_-mediated signaling pathways^40^. With the genetically encoded reporters HyPer for H_2_O_2_ and of Grx1-roGFP2 for the glutathione redox buffer we now confirm this hypothesis and provide quantitative details on the spatial/temporal development of H_2_O_2_ in response to IR and UV light. One important observation is that the concentration of H_2_O_2_ increases not only in the nucleus but also in the cytoplasm; this occurs after X-ray as well as after UV-light irradiation. It is interesting to note that H_2_O_2_ increases much faster in response to UV micro-irradiation than to X-ray exposure. Even though we cannot compare the doses of both treatments the data still suggest that the molecular mechanisms, which are underling the generation of H_2_O_2_ in response to both treatments, are fundamentally different. This conclusion is consistent with the fact that X-ray irradiation is sufficiently energetic for the hydrolysis of water while that of UV-A light is not. UV-A light- triggered H_2_O_2_ generation, which is evident in the nucleus and in the cytosol, could alternatively originate from endogenous photosensitizers, which are made responsible for the generation of H_2_O_2_ and DNA damage in the nucleus by UV-A light^28^,^29^; similar UV-A absorbing photosensitizers may also be present in the cytosol. The micro-irradiation experiments also underline a compartmentation of the H_2_O_2_ signal in either nucleus or cytoplasm; H_2_O_2_ does neither leak from the nucleus into the cytoplasm or vice versa. This is in agreement with data, which report that membranes are a significant diffusion barrier for H_2_O_2_^41^.

A recent report had shown that irradiation of A549 and HEK293 cells with 1 Gy X-ray, a dose which is commonly used in cancer therapy, caused an activation of the human intermediate conductance Ca^2+^ activated K^+^ (hIK) channel^16^. As an explanation for the radiation-stimulated increase in K^+^ channel activity it was speculated that IR may trigger an early evolution of H_2_O_2_ and that the latter activates hIK channels via an elevation of the concentration of cytosolic Ca^2+^ (Ca^2+^_cyt_)^16^. The present data confirm this hypothesis. A direct exposure to 1 Gy X-ray caused a small but robust increase in the H_2_O_2_ concentration in A549 and HEK293 cells. The mean increase of this transient H_2_O_2_ signal is roughly equivalent to what can be induced in the same cells with external H_2_O_2_ in the low micromolar range. The prediction that micromolar concentrations of H_2_O_2_ in the external medium should then activate the Ca^2+^ sensitive hIK channels in A549 cells is confirmed by experimental data. Here we find that 3 μM H_2_O_2_ in the external buffer indeed causes an elevation of Ca^2+^_cyt_. This is consistent with the finding that the same treatment causes in A549 cells an activation of hIK channels^16^. The fact that an elevation of the cytosolic H_2_O_2_ concentration has no effect on the membrane conductance of HEK293 cells is consistent with data, which show that hIK channels are not functionally expressed in HEK293 cells. Collectively the data suggest that 1 Gy X-ray is generating a genuine increase in the concentration of H_2_O_2_ in cells. It is difficult to really quantify the excursion in the cellular H_2_O_2_ concentration in response to 1 Gy X-ray but from an *in vitro* calibration of the HyPer sensor we can estimate that the peak will be in the low nanomolar range. This means that the differential response of the membrane conductance in different cell lines to IR is not caused by differences in the generation of the primary H_2_O_2_ signal; a radiation triggered increase in conductance is limited to cells which express hIK channels.

The transient nature of the HyPer signal in combination with monitoring of the redox state of glutathione, the dominant cellular buffer of radicals, shows that H_2_O_2_ is efficiently scavenged both in the cytosol and in the nucleus. The actual concentration of H_2_O_2_ must be seen as the product of its production and buffering. This interplay of H_2_O_2_ production and buffering can be an explanation for the rather slow increase in a measurable elevation of H_2_O_2_ and hIK channel activity after irradiation with 1 Gy^16^. H_2_O_2_, which is initially produced in response to IR, is presumably rapidly buffered; in this way it does not cause a measurable change in H_2_O_2_ concentration. Significant elevation of H_2_O_2_ in the cells is occurring once the buffer is exhausted. This resulting rise in the free concentration of H_2_O_2_ is apparently able to trigger Ca^2+^_cyt_-mediated signaling cascades in the cytosol. The exact mechanism by which this occurs cannot be extracted from the present data. But several other studies have already reported a causal link between H_2_O_2_ and Ca^2+^_cyt_ signaling. One example is the activation of store-operated Ca^2+^ channels by hydrogen peroxide^42^ but also an inhibition of these channels by H_2_O_2_ is reported^43^. In another context it was found that H_2_O_2_, which was formed under UV-A stress, stimulated phospholipase C activity^40^. All these H_2_O_2_ sensitive signaling pathways can in principle be involved in triggering or tuning Ca^2+^_cyt_ oscillations and the consequent wide range of physiological reactions.

## Methods

### Plasmids

Plasmids were obtained from Alex Costa, University of Milan, Italy (pHyPer-cyto) Vsevolod Belousov, Institute of Bioorganic Chemistry, Moscow, Russia (pSypHer-cyto) and Andreas Meyer, University of Bonn, Germany (pLPCX-Grx1-roGFP2).

### Cell culture

Cells were cultivated under standard conditions with 37 °C ambient temperature and 5% CO_2_. A549 and HEK293 cells were kept in Dulbecco’s Modified Minimal Essential Medium 1:1 Ham’s F-12 (DMEM Ham’s F-12) medium with stable glutamine, supplemented 10% fetal calf serum (FCS) and 1% penicillin/streptomycin. A549 cells were cultivated in media additionally supplemented 1% non-essential amino acids (NEAA).

Cells at about 60% confluency, which translates in ca. 200.000 cells on a 25 mm cover slip, were transiently transfected with plasmid DNA and TurboFect (Thermo Fisher Scientific Inc., Waltham, MA, USA), GeneJuice (Novagen, Merck KGaA, Darmstadt, Germany), or Lipofectamine (Life Technologies GmbH, Darmstadt, Germany) according to the manufacturers’ protocols. Cells were used ca. 24 h after transfection for microscopic analysis. The round cover slips where therefore fixed on the bottom of a stainless steel, custom-made incubation chamber, which is able to hold several 100 μl of buffer.

### Patch clamp recordings

Membrane currents of cells were recorded as described previously^16^ with a portable patch-clamp device (port-a-patch, Nanion, Munich, Germany), the EPC-9 amplifier (HEKA Electronics, Lambrecht, Germany) and PatchMaster software (HEKA, Lambrecht, Germany). The intracellular solution contained (in mM) 50 KCl, 10 NaCl, 60 KF, 10 Hepes/KOH pH 7.2, 1 EGTA (sufficient to have no free Ca^2+^ at the beginning of the experiment), Sorbitol was used to adjust the osmolarity to 285 mOsmol/kg. The extracellular solution contained 140 NaCl, 4 KCl, 1 MgCl_2_, 2 CaCl_2_, 10 Hepes/NaOH pH 7.4; Sorbitol was used to adjust the osmolarity to 300 mOsmol/kg. Cells were sealed in solution containing 80 NaCl, 3 KCl, 10 MgCl_2_, 35 CaCl_2_, 10 Hepes/NaOH pH 7.4, Sorbitol was used to adjust the osmolarity to 300 mOsmol/kg.

### H_2_O_2_ imaging

Single-cell imaging was performed at room temperature on a Leica confocal system TCS SP5 II with the software LAS AF Version 2.60 (Leica Microsystems CMS GmbH, Heidelberg). Images were acquired and UV-laser micro-irradiation was performed with a 40x (1.3 NA) oil- immersion objective. Cells were grown on 25 mm round glass coverslips (No. 1.0) and measured in PBS (Sigma-Aldrich GmbH, Taufkirchen, Germany), if not mentioned otherwise. HyPer, SypHer and Grx1-roGFP2 were sequentially excited with a 405 nm diode and with an argon laser at 488 nm. Images (512 × 512 pixels in size) were acquired with a 12 bit HyD detector at 500–550 nm. For HyPer and SypHer the background subtracted (selected ROIs at cell-free positions) ratio 488 nm/405 nm (F_488/_F_405_) is plotted for all experiments. For Grx1-roGFP2 the ratio was calculated by division of the background subtracted fluorescence intensity 405 nm/488 nm (F_405_/F_488_). Image analysis was performed with the open source software FIJI (http://fiji.sc).

The HyPer and Grx1-roGFP2 sensors were transiently expressed in A549 or HEK293 cells and analyzed with respect to their stability. In long-term recordings it occurred that the respective ratios of F_488/_F_405_ for Hyper and F_405_/F_488_ for Grx1-roGFP2 showed in un-stimulated control cells no appreciable variations. The background variance in the ratios was smaller than 5*10^−6^ for Grx1-roGFP2 and 5*10^−4^ for HyPer. Only after addition of extracellular H_2_O_2_ the respective ratios increased.

### Ca^2+^ and redox buffer imaging

The sensor Fluo-4 was loaded into A549 cells by incubating cells, which were grown on cover slips, for 30 min in buffer (140 mM NaCl, 4 mM KCl, 1 mM MgCl_2_, 5 mM Sorbitol, 10 mM HEPES, 2 mM CaCl_2_, pH 7.4.) containing 1 μM Fluo-4 AM (Life technologies, Carlsbad, California, USA). The dye was subsequently removed by washing cells with dye free buffer. Cells were then imaged in the equatorial plane every 5 sec on a Leica TCS SP5 II confocal microscope (Leica, Heidelberg, Germany) using a HCX PL APO CS 40.0 × 1.30 OIL oil immersion lens. The dye was excited with a 488 nm argon laser and the emission sampled at 505–560 nm. The fluorescence of cells was first monitored for 3 min to obtain a measure for the background fluorescence. The incubation buffer was then replaced by the same buffer with 3 μM H_2_O_2_. At the end of a recording 5 μM Ionomycin (Sigma Aldrich, Taufkirchen, Germany) was added in order to obtain the maximal fluorescence of the Fluo-4 dye. To account for drifts in the background signal the latter was subtracted from the cell-related signal.

### Cell irradiation

For laser micro-irradiation (m.i.) a continuous wave 405 nm diode laser was focused via a 40x (1.3 NA) oil-immersion objective. The power of the laser beam was about 450 μW at the sample plane, which was measured by a UV dosimeter (Powermeter PM100D with S130C sensor; Thorlabs, Newton, New Jersey, USA). The laser beam was repeatedly scanned in the region of interest (ROI) with a pixel dwell time of 2.54 μsec. The resulting deposed laser energy in the ROI was obtained by varying the spot size of the ROI as well as the irradiation time. Predefined ROIs either in the cytoplasm or in the nucleus were exposed to a high intensity (0.5–4.5 μJ/ μm^2^) of 405 nm UV-laser.

Live microscopic experiments were performed with cells on 40 mm glass coverslips on a custom-build microscope, which is coupled to an X-ray source (GSI Helmholtzzentrum für Schwerionenforschung, Darmstadt, Germany). Imaging was performed with an Olympus IX71 microscope using a 60x (1.2 NA) water-immersion objective and additional 1.6x Optovar in combination with an Andor IXON 888 EMCCD operating under Andor IQ 1.10.5 software. The setup is equipped with an X-ray tube (Isovolt, GE Sensing & Technologies, Ahrensburg, Germany), operated at 35 kV and 80 mA (dose rate 32 Gy/min ± 10% or 35 kV and 20 mA (dose rate 8.6 Gy/min ± 10%), filtered with a 0.5 mm aluminum sheet. The applied dose was controlled with a PTW D14 dosimeter (PTW, Freiburg, Germany).

## Additional Information

**How to cite this article**: Gibhardt, C. S. *et al.* X-ray irradiation activates K^+^ channels via H_2_O_2_ signaling. *Sci. Rep.* **5**, 13861; doi: 10.1038/srep13861 (2015).

## Supplementary Material

Supplementary Information
