# Supplementary material for: X-ray irradiation activates K+ channels via H2O2 signaling
Source: Sci Rep. 2015 Sep 9;5:13861. doi: 10.1038/srep13861 (PMC4642570; doi:10.1038/srep13861)
Supplement: Supplementary Information [file srep13861-s1.pdf]

Supplementary information

X-ray irradiation activates K^+ channels via H_2O_2 signaling

Christine S. Gibhardt[1], Bastian Roth[1], Indra Schroeder[1], Sebastian Fuck[1], Patrick Becker[1], Burkhard Jakob[2], Claudia Fournier[2], Anna Moroni[3], Gerhard Thiel[1]

[1]Department of Biology, Membrane Biophysics, Technische Universität Darmstadt, Schnittspahnstrasse 3, 64287 Darmstadt, Germany
[2]Department of Biophysics, GSI Helmholtzzentrum für Schwerionenforschung GmbH, Planckstrasse 1, 64291 Darmstadt, Germany
[3]Department of Biosciences and CNR IBF-Mi, Università degli Studi di Milano, Via Celoria 26, 20133 Milano, Italy

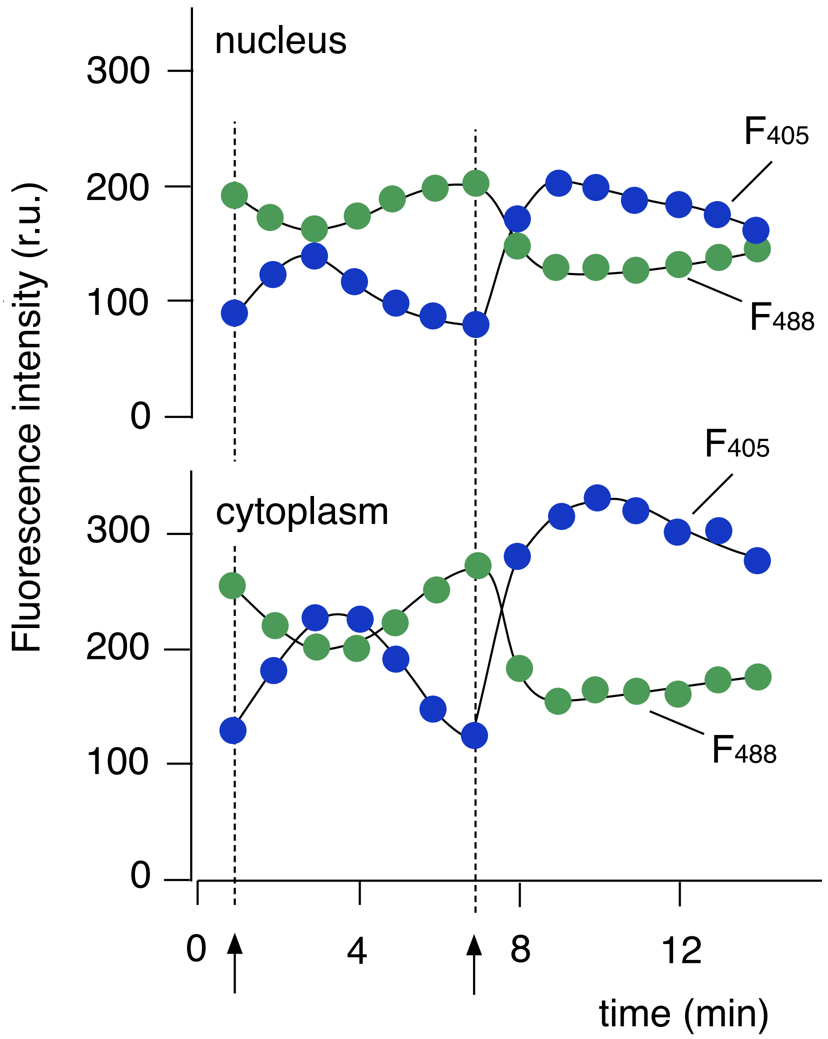

Figure S1: Fluorescence changes of HyPer sensor in response to H_2O_2. Fluorescence intensity in relative units (r.u.) at 488 nm (F_{488}, green symbols) and 405 nm (F_{405}, blue symbols) from HyPer in nucleus (top) and cytoplasm (down) in a HEK293 cell in response to different concentrations of external H_2O_2. The arrows/dotted lines indicate the increase of the external H_2O_2 concentration from 0 to 20 µM (left arrow) and 100 µM (right arrow). The data were used to calculate the F488/F405 ratios in Fig. 1A.

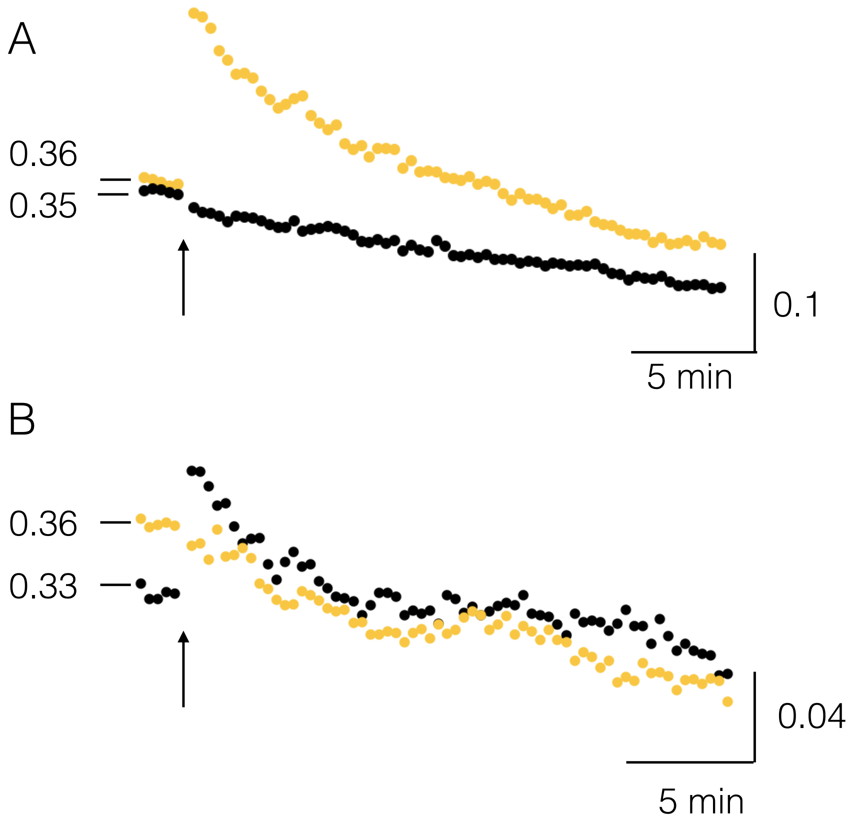

Figure S2: UV-laser micro-irradiation triggers compartment specific rise in H_2O_2. UV-micro-irradiation induced change in HyPer ratio in nucleus (orange) and cytosol (black) from cells shown in Fig. 2. Data in (A) correspond to recordings in Fig.2A;B and show HyPer response to irradiation of the nucleus. Data in (B) correspond to experiment in Fig. Fig 2. B,D with micro-irradiation of the cytosol. Numbers on traces denote the ratio at start of recording. Irradiation was achieved with a 405 nm laser (3 µJ/µm^2) at time indicated by arrows. Vertical calibration bars show ratio F_{488}/F_{405} for HyPer.

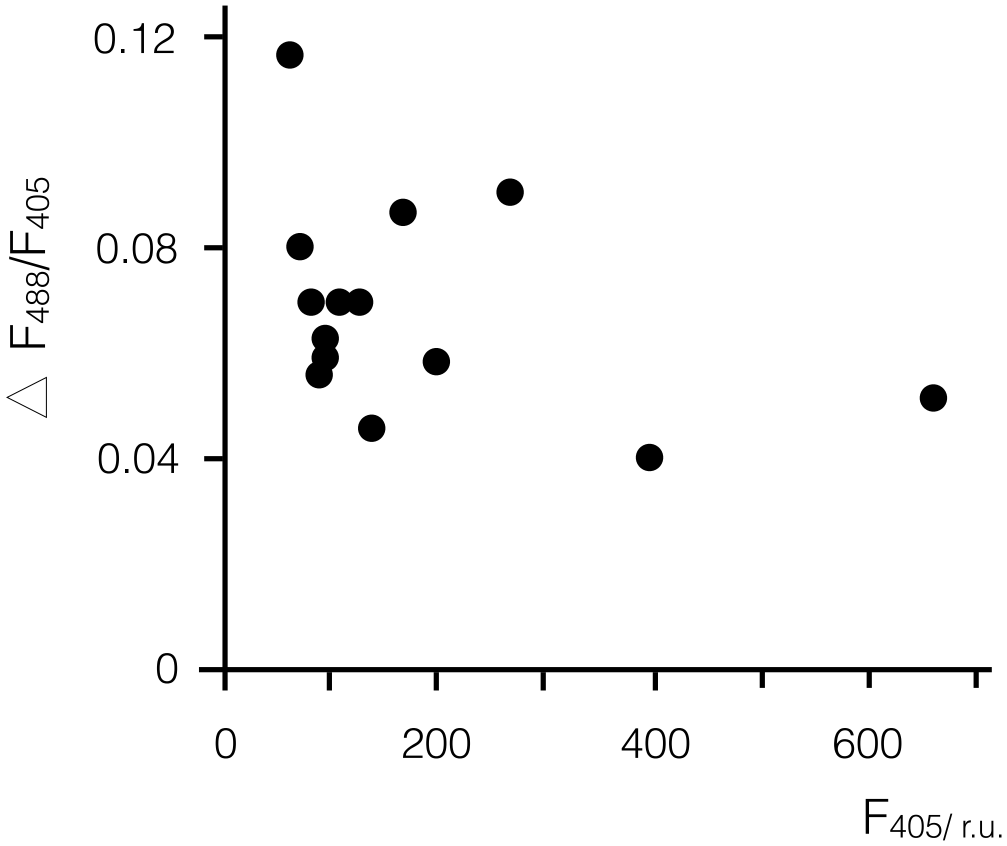

Figure S3: Increase in HyPer signal, which is evoked by UV-laser micro-irradiation, is not depending on HyPer concentration in cells.

Fifteen A549 cells, which were expressing H_2O_2 sensor HyPer, were micro-irradiated with a 405 nm laser (3 μJ/μm^2). The irradiation-triggered increase in the ratio F_{488}/F_{405} for HyPer is plotted as a function of the absolute fluorescence intensity at 405 nm; the latter is an indirect measure of the HyPer concentration in each cell investigated.

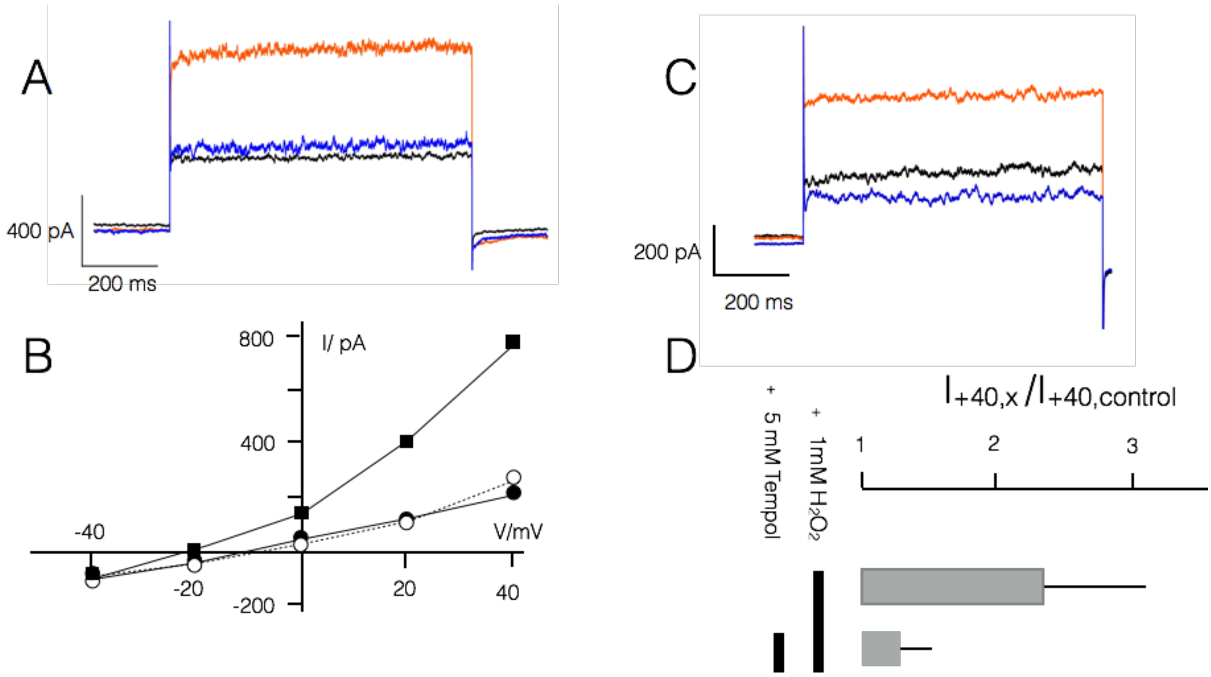

Figure S4: Irradiation of A549 cells and H_2O_2 cause increase in hIK channel conductance. (**A**) Exemplary current responses of one A549 cell to voltage pulse from -80 mV to +40 mV and back to the initial voltage. Currents were recorded 4 min before (black) and 6 min (orange) after start of X-ray irradiation with 10 Gy. The currents were again recorded 6 min after treating the cell with 300 nM TRAM-34 (blue). Current values were sampled after decay of transient capacitive current. (**B**) Current/voltage (I/V) relations from the same cell measured prior (closed circle), 6 min after start of irradiation (filled squares) and 6 min after addition of TRAM-34 (open circle). Note that irradiation augments a negative shift of the reversal voltage (a hyperpolarization), which is reverted by the hIK blocker. Currents were measured as in (A) with pulse protocol from a holding voltage (-60 mV) to test voltages between −80 mV and +60 mV in 20 mV steps and a post pulse to -80 mV. (**C**) Exemplary current responses of a A549 cell to voltage pulse from -80 mV to +40 mV. Currents were recorded 3 min before (black) and 1 min (orange) after addition of 1 mM H_2O_2 to the external bath solution. The currents were again recorded 1 min after adding 5 mM of the redox scavenger Tempol to the intracellular medium (blue). (**B**) Relative current changes at +40 mV induced by 1 mM H_2O_2 and subsequent addition of 5 mM Tempol. The relative currents were obtained by dividing the currents at +40 mV in the presence of a stimulus ($I_{+40,x}$) e.g. H_2O_2 and H_2O_2 plus Tempol, by the control current recorded prior to treatment ($I_{+40,control}$). Data are mean ± s.d. from 4 cells. In 3 cells Tempol was perfused directly into the cytosol in one cell it was added externally.
